# Using domain adaptation for classification of healthy and disease conditions from mobile-captured images of standard 12-lead electrocardiograms

**DOI:** 10.1038/s41598-023-40693-6

**Published:** 2023-08-28

**Authors:** Vadim Gliner, Vladimir Makarov, Arutyun I. Avetisyan, Assaf Schuster, Yael Yaniv

**Affiliations:** 1 Computer Science Department, Technion-IIT, Haifa, Israel; 2 System Programming Lab, Novgorod State University, Veliky Novgorod, Russia; 3 Ivannikov Institute for System Programming of the Russian Academy of Sciences, Moscow, Russia; 4 Laboratory of Bioenergetic and Bioelectric Systems, Biomedical Engineering Faculty, Technion-IIT, Haifa, Israel

**Keywords:** Physiology, Cardiology

## Abstract

12-lead electrocardiogram (ECG) recordings can be collected in any clinic and the interpretation is performed by a clinician. Modern machine learning tools may make them automatable. However, a large fraction of 12-lead ECG data is still available in printed paper or image only and comes in various formats. To digitize the data, smartphone cameras can be used. Nevertheless, this approach may introduce various artifacts and occlusions into the obtained images. Here we overcome the challenges of automating 12-lead ECG analysis using mobile-captured images and a deep neural network that is trained using a domain adversarial approach. The net achieved an average 0.91 receiver operating characteristic curve on tested images captured by a mobile device. Assessment on image from unseen 12-lead ECG formats that the network was not trained on achieved high accuracy. We further show that the network accuracy can be improved by including a small number of unlabeled samples from unknown formats in the training data. Finally, our models also achieve high accuracy using signals as input rather than images. Using a domain adaptation approach, we successfully classified cardiac conditions on images acquired by a mobile device and showed the generalizability of the classification using various unseen image formats.

## Introduction

Interpretation of 12-lead electrocardiogram (ECG) recordings is the main clinical tool for detection of cardiac conditions, including both morphological and arrhythmogenic disorders. Today, while 12-lead ECG can be performed in any clinic, the recordings can only be analyzed by experienced cardiologists. The recent development of large publicly available 12-lead ECG datasets, along with the rapidly advancing machine learning (ML) -based algorithmic paradigms that require large datasets, have motivated attempts to design ML-based 12-lead ECG interpretation^1–4^. Automated and accurate 12-lead ECG analysis is likely to provide higher-precision clinical evaluation and enable worldwide population screening.

While ML algorithms may advance the field of automated12-lead ECG interpretation, they are not yet being used by practitioners because they fall short of providing a solution to several major clinical requirements. (i) In many clinics, ECG recordings are printed on paper or exist in image format, and are later analyzed by an expert. However, most ML-based 12-lead ECG interpretation algorithms are designed for digitized signals as their input. Considering that 300 million paper ECGs are obtained annually worldwide^5^, algorithms assuming input formatted as a digital signal (rather than as a printout or a digital image) miss huge potential datasets. Such vast sources are important for a specific class of modern ML models known as deep neural networks (DNNs)^6^ which require very large sets of training data. (ii) While it would be most sensible to digitize the ECG machine printout using the highly-available mobile cameras (e.g., a smartphone cameras), images captured by a mobile device comprise various artifacts and tend to be tilted, skewed, and crumpled, and include shadowing. This adds a significant processing challenge to automatic interpretation of the resulting image. (iii) Different vendors of ECG machines use different formats to plot the 12-lead ECG signal (see Fig. S1); tens or even hundreds of different formats are in use worldwide. To train an automatic ECG interpretation algorithm to be able to analyze all formats, massive training sets would have to be obtained plotted in each format, which is an unfeasible task. (iv) Training high-capacity DNNs necessitates huge volumes of labeled data, which would require extensive labor to collect and validate. Furthermore, labeling requires the effort of expert cardiologists and is thus very costly. As a result, the volume of publicly available, high-quality labeled data that can be used to develop accurate ML models, is limited.

Domain adaptation is a field that was developed for computer vision, with the goal of training a neural network on a source dataset and securing adequate accuracy on a target dataset that has a significantly different distribution than the source dataset^7^. Importantly, domain adaptation can be performed in an unsupervised manner, thereby reducing the dependence of the solution on the availability and labor of expert cardiologists^7^. Domain adaptation techniques have already been shown to improve the state of the art in medical applications^8^. To make use of these ideas we suggest in this work to use domain adversarial neural network structure, called ECG-Adversarial, and a tailored training method.

The main objective of this work was to show how domain adaptation techniques can overcome the above-mentioned challenges which limit automatic 12-lead ECG interpretation of mobile images captured in clinical environments. We used domain adaptation techniques to achieve high accuracy on tested images captured by a mobile device, without training on such images with their corresponding labels. The network accuracy was improved on inclusion of a small number of unlabeled samples of unknown formats in the training data. Finally, the models also achieved high accuracy using ECG signals rather than images as input.

## Results

### General approach

**Figure 1:**
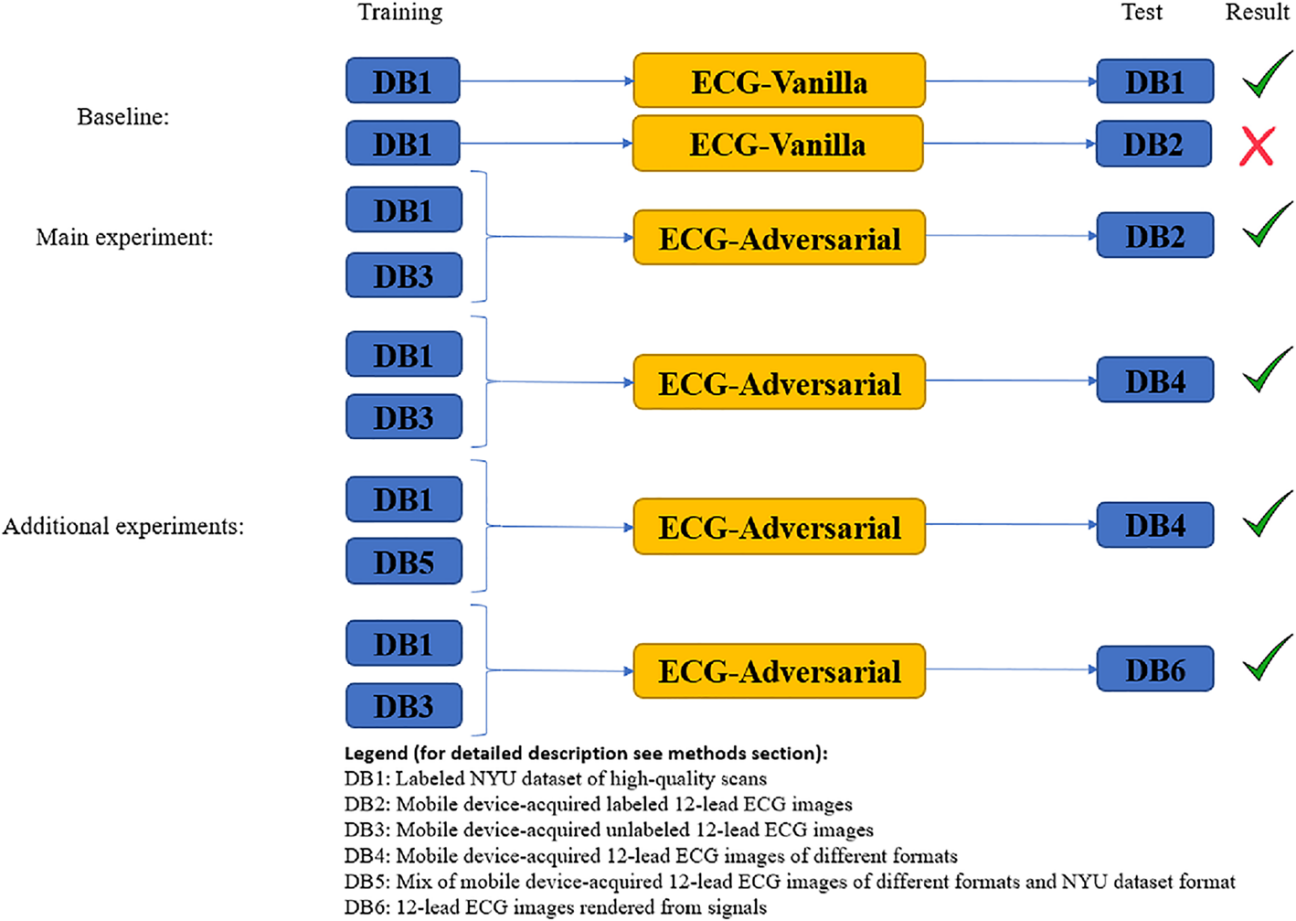
Schematic diagram of overall pipeline of this work.

The results section presents three sets of experiments (see Fig. 1). In the baseline experiment, our published ECG-Vanilla net^4^ was tested with either the labeled NYU dataset of high-quality scans (https://education.med.nyu.edu/ecg-database/app) or unlabeled mobile device-acquired 12-lead ECG images. Because only the scanned 12-lead ECG images were labeled, they were vital for achieving high accuracy in heart disease classification. These experiments aimed to assess the feature-extraction capability of ECG-Vanilla and to justify the adversarial approach. As expected, ECG-Vanilla showed poor performance when tested on images from different distributions and/or different formats than those of the NYU dataset. The main experiment tested ECG-Adversarial on mobile device-acquired 12-lead ECG images. Additional experiments aimed to assess the performance of ECG-Adversarial net on images generated from signals and on various unseen formats.

### Detection of multiple heart conditions from 12-lead ECG images using the convolutional neural network ECG-Vanilla

**Figure 2:**
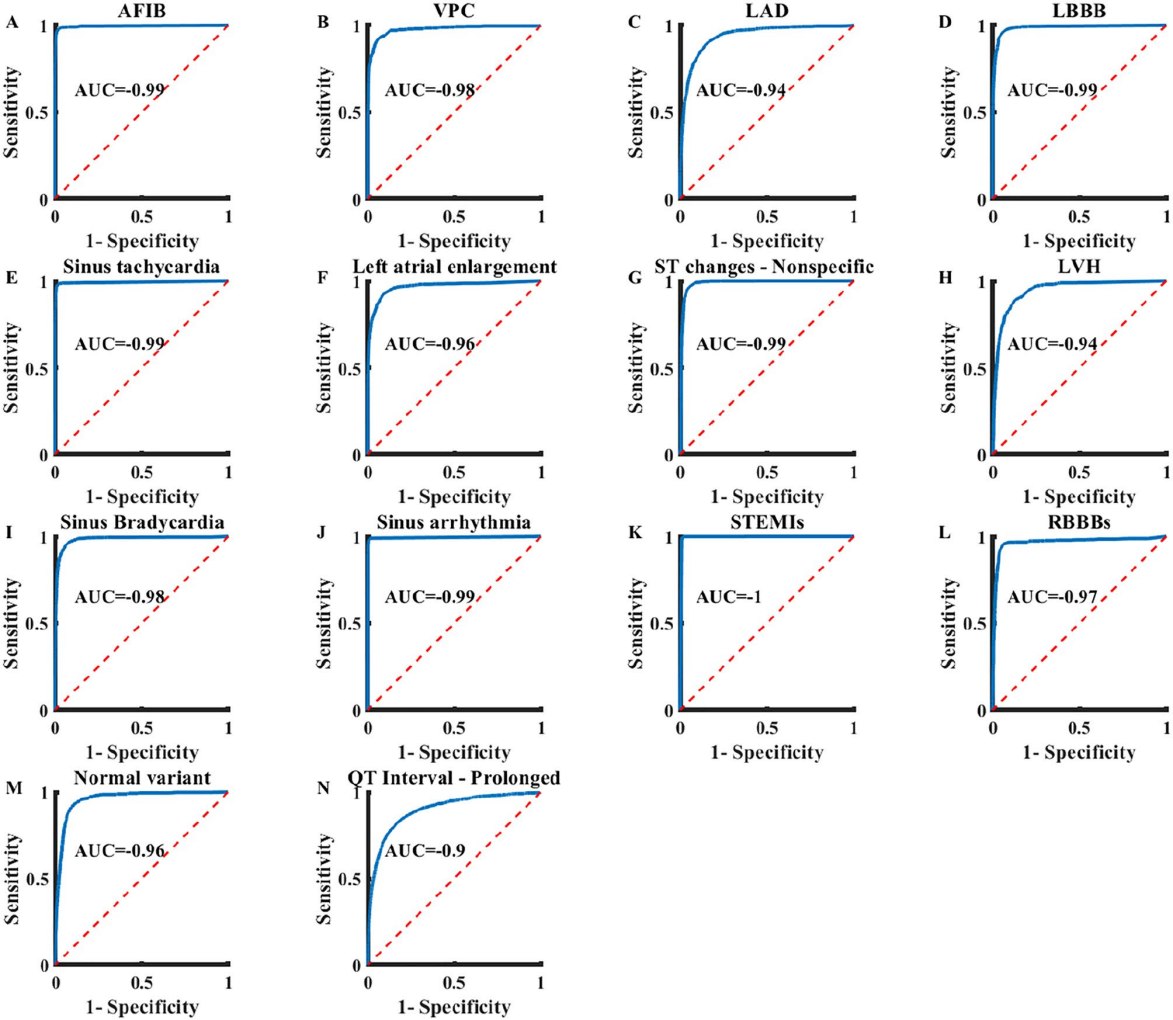
The receiver operation characteristic area under the curve (ROC-AUC) of cardiac condition identification by ECG-Vanilla. (**A**) Atrial fibrillation (AFIB) 0.99, (**B**) Premature ventricular contraction (VPC) 0.98, (**C**) Left-axis deviation (LAD) 0.97, (**D**) Left bundle branch block (LBBB) 1.0, (**E**) Sinus tachycardia 0.99, (**F**) Left atrial enlargement (LAE) 0.98, (**G**) ST changes—Nonspecific 0.9, (**H**) Left ventricular hypertrophy (LVH) 0.99, (**I**) Sinus bradycardia 0.99, (**J**) Sinus arrhythmia 0.96, (**K**) ST-elevation due to myocardial infarction 0.94, (**L**) Right bundle branch block (RBBB) 0.99, (**M**) Normal variant 0.96, (**N**) Prolonged QT interval 0.94.

In this section we first present 12-lead ECG analysis results using a convolutional network, named ECG-Vanilla^4^, trained, and tested using perfectly scanned images from the NYU dataset. ECG interpretations must bear capacities to identify and differentiate between cardiac comorbidities, as well as rare diseases. To this end, we developed ECG-Vanilla with a generic per-disease architecture that is separately trained for binary identification (see “Methods”). Table 1 shows the accuracy and F_1_ score (the harmonic mean of the positive predictive value) for detection of 14 diseases, with the cardiologist labeling taken as the ground truth. Both training and test sets with the same distributions (i.e., scanned images) were taken from the NYU dataset. The detection accuracy and F_1_ score of the network for the test set were 0.83–0.99 and 0.82–0.99, respectively, depending on the disease type and the amount of training data available for that disease. The network achieved ROC-AUC > 0.9 for all heart conditions (Fig. 2). The class weighted average ROC-AUC was 0.97. Supplementary Table S1 presents more statistical measurements.

**Table 1:** ROC-AUC and F_1_ of ECG-Vanilla. The NYU dataset was divided to a training set (83%), internal validation dataset (2%) and a hidden test set (15%). A hidden test set is a set of examples not included in any part of the training.

Category	ROC-AUC training set	ROC-AUC test set	F_1_ test set
Atrial fibrillation	0.99	0.98	0.98
Premature ventricular contraction	0.95	0.93	0.93
Left axis deviation	0.95	0.95	0.95
Left bundle branch block	1.0	0.99	0.99
Sinus tachycardia	1.0	0.99	0.99
Left atrial enlargement	0.95	0.95	0.95
ST changes	0.84	0.83	0.82
Left ventricular hypertrophy	0.97	0.96	0.96
Sinus bradycardia	0.99	0.98	0.98
Sinus arrhythmia	0.98	0.92	0.92
ST elevation due to myocardial infarction	0.91	0.87	0.87
Right bundle branch block	0.96	0.95	0.95
Normal variant	0.93	0.91	0.91
Prolonged QT interval	0.95	0.88	0.88
Average	0.96	0.94	0.93

To compare the performance of ECG-Vanilla to that of a state-of-the-art net, the same training routine (see “Methods” section) was used for both ECG-Vanilla and ResNet18. ECG-Vanilla was slightly superior to ResNet18 (Table S2).

### Detection of multiple disorders from unlabeled mobile device-acquired 12-lead ECG images with distortion, using a convolutional network ECG-Adversarial

ECG-Vanilla proved very efficient in detecting tested diseases, as shown by the high accuracy and high F_1_ scores (Table 1). However, when tested on images from the same format, but that were captured by mobile device (to mimic clinical conditions), its performance was relatively poor (Table 2). To improve performance, we designed a domain-adversarial neural network called ECG**-**Adversarial, comprising both a label predictor and a domain classifier. In the context of this section, domain adversarial training attempts to extract features to accurately identify cardiac conditions (label predictor), but at the same time, it intentionally deteriorates the ability of the domain classifier to determine if the example is an original ECG image or an ECG image with mobile device acquisition distortion. As a result, the extracted features are only those which do not rely on a perfectly scanned image, thus training the network to find features that enable it to also diagnose mobile-captured images.

**Table 2:** Comparing the accuracy of ECG-Vanilla and ECG-Adversarial using three different training configurations.

Category	ROC-AUC	ROC-AUC	ROC-AUC	ROC-AUC
	ECG-Vanilla	ECG-Adversarial no augmentation, dropout = 0.15, epoch of start training the domain head = 3	ECG-Adversarial with augmentation, dropout = 0.15, epoch of start training the domain head = 3	ECG-Adversarial with augmentation, dropout = 0.25, epoch of start training the domain head = 5
Atrial fibrillation	0.37	0.80	0.94	0.96
Premature ventricular contraction	0.51	0.79	0.74	0.82
Left axis deviation	0.81	0.84	0.91	0.88
Left bundle branch block	0.61	0.91	0.94	0.95
Sinus tachycardia	0.50	0.87	0.83	0.85
Left atrial enlargement	0.49	0.87	0.85	0.95
ST changes	0.60	0.72	0.88	0.88
Left ventricular hypertrophy	0.91	0.92	0.85	0.93
Sinus bradycardia	0.93	0.92	0.98	0.95
Sinus arrhythmia	0.47	0.78	0.8	0.84
ST elevation, myocardial infarction	0.54	0.72	0.73	0.89
Right bundle branch block	0.50	0.80	0.82	0.94
Normal variant	0.86	0.87	0.84	0.95
QT Interval, prolonged	0.50	0.82	0.95	0.96
Average	0.62	0.83	0.86	0.91

**Figure 3:**
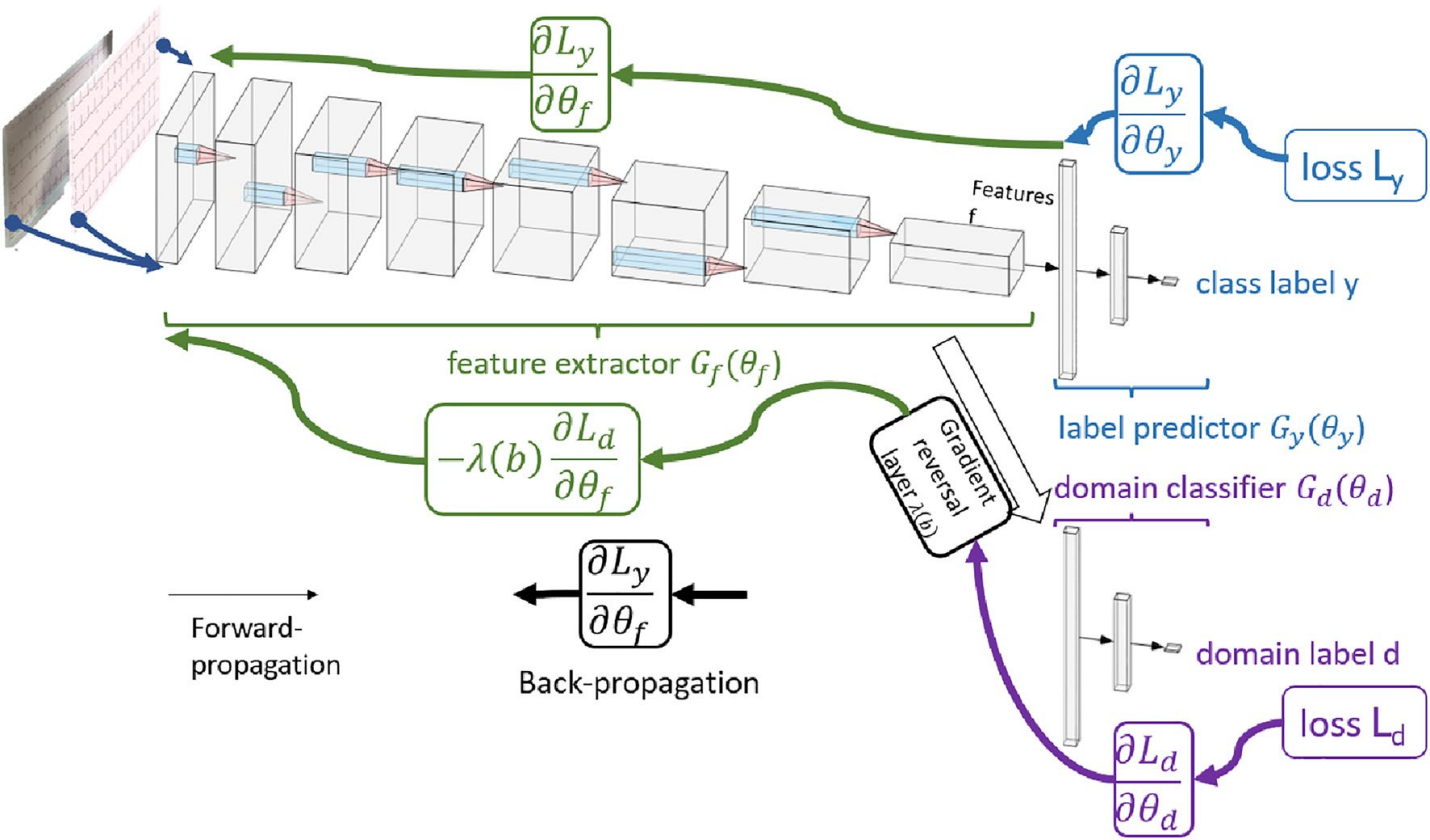
Architecture of ECG-Adversarial. Two datasets were used for training and testing: the New York University (NYU) dataset containing perfectly scanned and labeled 12-lead ECG images (NYU dataset), and a dataset containing mobile-captured 12-lead ECG images. The latter was generated by printing the NYU dataset images on paper, using a mobile camera to capture images with various artifacts, and splitting the movie into frames. The input 12-lead ECG images from the NYU dataset are propagated forward and backward (blue and green, respectively) through the feature extractor. The same images with additional images captured by a mobile device, are also propagated forward and backward through the domain classifier (purple and green, respectively). When they are backpropagated through the domain classifier, adaptive gradient reversal is applied, essentially causing the construction to ignore domain-specific features^9^. The input from the second dataset, namely, mobile-captured 12-lead ECG images, is propagated forward and backward (purple and green, respectively) through the domain classifier path only. When they are backpropagated through the domain classifier (purple), gradient reversal is applied^9^. As a result of this domain adversarial method, the feature extractor is forced to ignore domain-specific features. In our case, domain-specific features either belong only to “clean” images that were not mobile-captured or are format-specific. Thus, the domain adversarial method allows the extractor to focus on features that better generalize to other domains (e.g., other 12-lead ECG formats and/or 12-lead ECG mobile-captured images), even though it was not trained using data from these domains.

The proprietary format used for plots generated by an ECG machine of a specific vendor can include dozens of features, such as grid scale, grid color, lead beginning and ending with calibration plots, lead placement, and others. In ML jargon, such a format is called a domain, where the challenge is to transfer learnt analysis capabilities from a domain where a lot of high-quality, labeled data is available to domains where only poor-quality, perhaps unlabeled training data is available. Another type of domain consists of images of 12-lead ECG plots acquired using mobile devices. Once again, the challenge here is to enable accurate analysis of data from that domain while the training is carried out using data from the domain of high-quality, scanned images with no distortions and artifacts. To meet both challenges, ECG-Adversarial makes use of unlabeled 12-lead ECG samples from target domains (various formats, mobile camera-captured images) together with samples from the high-quality, labeled data domain. See high-level description in Fig. 3.

In our experiments, ECG-Adversarial was trained on a dataset comprising 79,226 scanned and labeled 12-lead ECGs from the NYU School of Medicine Emergency Care Database and a dataset comprising 39,613 unlabeled mobile device-captured 12-lead ECG images. The test here includes only mobile captured labeled 12-lead ECG images.

The first configuration tested did not include augmentation (a method to generate augmented training images), and used low dropout (a method for avoiding overfitting and achieving regularization), with early disturbance (training the domain classifier starting from the third epoch). The second configuration included augmentation and low dropout with early disturbance. Although one may assume that augmentation of the input should always improve system performance and reduce overfitting, some data showed otherwise^10^. Application of augmentation resulted in minor performance degradations for specific cardiac conditions (e.g., sinus tachycardia, left ventricular hypertrophy and left atrial enlargement; Table 2—ROC-AUC without augmentation vs. ROC-AUC with augmentation, dropout = 0.15). Thus, we tested a third configuration with adjusted training hyperparameters (ROC-AUC with augmentation, dropout = 0.25, epoch of starting to train the domain head = 5).

**Figure 4:**
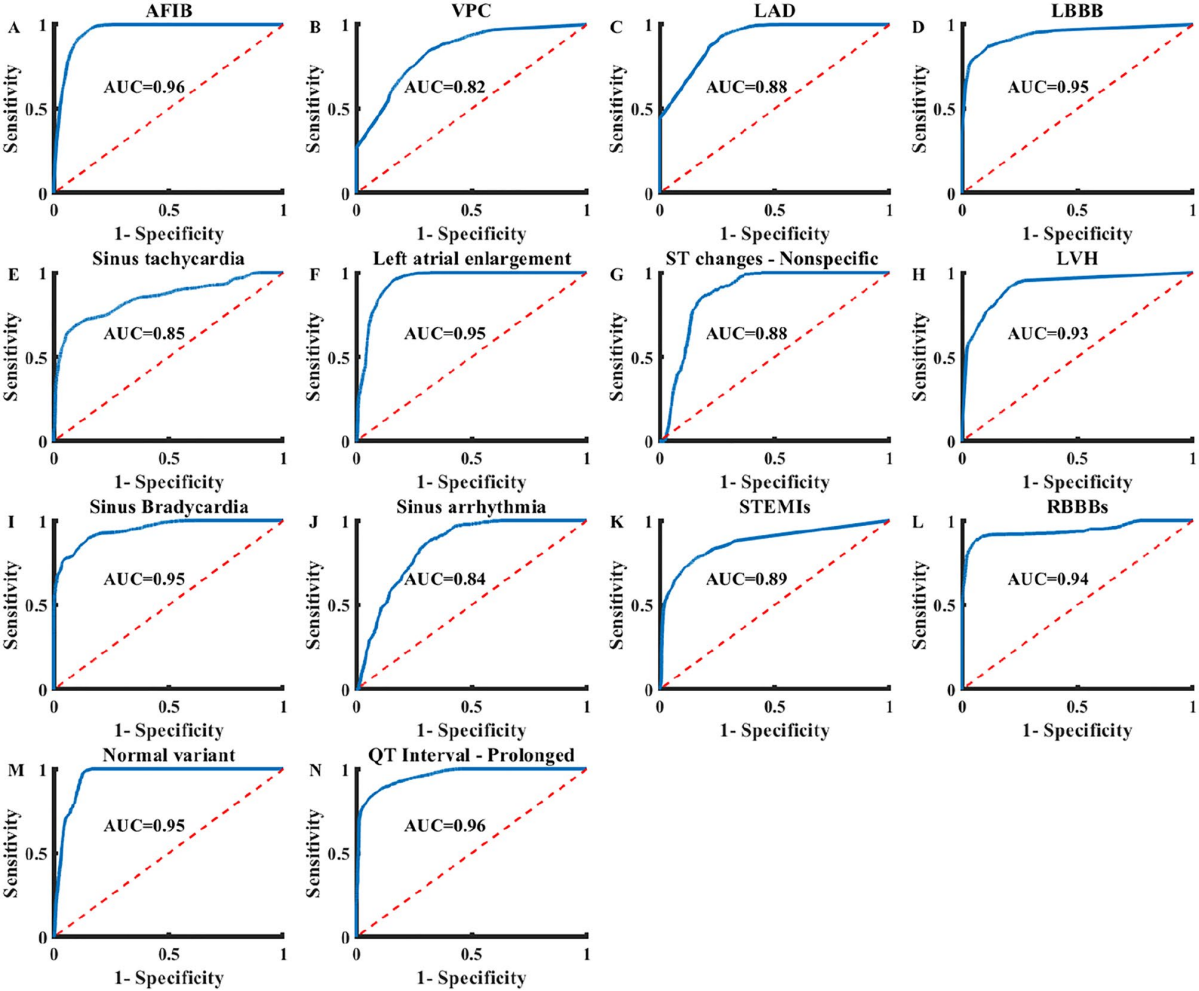
The receiver operation characteristic area under the curve (ROC-AUC) of ECG-Adversarial on a hidden test set of mobile device-acquired 12-lead ECG images. (**A**) Atrial fibrillation (AFIB), (**B**) Premature ventricular contraction (VPC), (**C**) Left-axis deviation (LAD), (**D**) Left bundle branch block (LBBB), (**E**) Sinus tachycardia, (**F**) Left atrial enlargement (LAE), (**G**) ST Changes—Nonspecific, (**H**) Left ventricular hypertrophy (LVH), (**I**) Sinus bradycardia, (**J**) Sinus arrhythmia, (**K**) ST-Elevation due to myocardial infarction, (**L**) Right bundle branch block (RBBB), (**M**) Normal variant, (**N**) Prolonged QT interval. Average ROC-AUC of the best configuration experimented with is 0.91.

The third configuration exhibited the best performance using domain adversarial training on mobile-acquired NYU ECG images (Table 2 and Fig. 4). The network achieved ROC-AUC > 0.82 for all heart conditions. The accuracy on the test set was 0.77–0.93, depending on the heart condition (Table S3). F_1_ score was 0.79–0.93 and the ROC-AUC was 0.84–0.96, depending on the heart condition.

Note that the train and test databases include both ideally scanned images and augmented ones. Thus, the system is compatible with both scanned photos as well as mobile device captures images.

### ECG-Adversarial detection of multiple disorders from an unseen format of 12-lead ECG images with mobile device acquisition distortion

12-lead ECG images do not have a uniform format, e.g., the lead placement on the image may vary or sometimes the long lead is not even present. In fact, obtaining training data with all existing formats is an unfeasible undertaking. To overcome this difficulty, we used our domain transfer approach, as in the case of mobile device-captured vs. perfectly scanned images, to train ECG-Adversarial to ignore format-specific features. To this end, the network domain classification head was fed with images of different formats and gradient reversal was used during backpropagation. We then tested whether ECG-Adversarial performs well on 12-lead ECG images with mobile device acquisition distortion whose format was not seen by the network during training (called here unseen formats). For testing, we used the third configuration of ECG-Adversarial, which achieved the highest average ROC-AUC scores in the mobile device capture experiments.

First, we tested ECG-Adversarial using mobile-acquired images of 12-lead ECG data with various unseen formats. The resulting ROC-AUC scores fell within the range of 0.94–1 (Fig. S2). Specifically, ROC-AUC for atrial fibrillation was 1.0, for sinus bradycardia was 0.99, for left-axis deviation was 0.99, for left ventricular hypertrophy was 1.0 and for sinus tachycardia was 0.94. Note that availability of data with various formats was restricted to this set of cardiac diseases, further emphasizing the importance and necessity of our methods: other formats using which there is no opportunity for training may suddenly be introduced as input during clinical operation.

One of the main advantages of the architecture and method proposed in this paper is that the results improve when, during training, or even later, during operation, the network is further retrained using several (unlabeled) examples from the target domain. Indeed, retraining the network after adding a few samples with unseen formats, improved the ROC-AUC score to 0.99–1 (Fig. S3).

### ECG-Adversarial detection of multiple disorders from 12-lead ECG signals

New ECG machines produced in recent years may provide a digital version of the 12 leads, essentially, time series of values stored as vectors, which we call here signals. It is relatively simple to turn signals into 12-lead ECG images. The reverse direction, however, is not straightforward; signal extraction, especially from images with artifacts, is a challenging task^11^.

To validate that our approach can use a hybrid environment where the input comprises both images and signals, we used input consisting of 12-lead ECG signals plotted on ECG paper background (see “Methods” section). Table S4 shows four performance assessments. In the first assessment, ECG-Adversarial was trained and tested using 12-lead ECG images with mobile device acquisition distortion. The average ROC-AUC score was 0.95. In the second assessment, ECG-Adversarial was trained similarly using 12-lead ECG images with mobile device acquisition distortion, but tested on images that were generated from signals from different databases (see “Methods”). Performance was similar or higher than reported above for unseen formats (see Table S4). The third assessment aimed to show that the accuracy of classification of ECG images with mobile device acquisition distortion is not negatively affected by addition of images generated from signals to the training set. The network was trained on a dataset consisting of both 12-lead ECG images with mobile device acquisition distortion and images that were generated from signals. The test was only performed on 12-lead ECG images with mobile device acquisition distortion. Diagnostic accuracy under these conditions was relatively close to that of the first assessment; average ROC-AUC was 0.90. In the fourth assessment, the network was trained on both 12-lead ECG images with mobile device acquisition distortion and images that were generated from signals and tested on images that were generated from signals. Under these conditions, the average ROC-AUC was similar to that of the third assessment, but substantially better than the accuracy achieved in the second assessment, where the training set did not include images that were generated from signals.

To summarize, our novel domain adversarial approach copes well with 12-lead ECG images captured by mobile device cameras as well as with digital signals plotted as an image. In addition, ECG-Adversarial network performance on signals can be further improved by including images generated from signals in the training process.

## Discussion

Standard 12-lead ECG is one of the most common tools used for cardiac disease diagnosis and is easily accessible. However, most recordings are available only in printed image formats. This work demonstrated that an ML-based ECG interpretation system can provide high accuracy analysis of mobile device-captured images of standard 12-lead printouts (Fig. 4). The proposed generic deep network can be used for data that is unlabeled, with illumination artifacts, geometric distortions, and even with 12-lead ECG formats that the network was not trained on. An ML-based system using our methods can be employed to provide a second opinion on the diagnosis of printed ECG, will enable storage of a labeled digital copy of the ECG recording for future use, and will enable massive, cost-effective screening of at-risk populations.

We first generated ECG-Vanilla trained and tested on samples from perfectly scanned images. This is the first time a “generic” architecture was used to provide high-accuracy analysis of ECG recordings to identify a large variety of cardiac conditions. However, ECG-Vanilla performance was relatively poor when tested on images captured by a mobile device camera (Table 2). Because under real clinical conditions^12^ a mobile device-captured image will contain artifacts and distortions that will make it very different from the images that ECG-Vanilla was trained on, this approach does not meet clinical requirements. To overcome this limitation, we proposed ECG-Adversarial, which uses transfer learning according to the model proposed by Ganin et al.^9^. The network includes a gradient reversal layer to ensure that features are domain-invariant. We used an adaptive parameter $\lambda$ to control and gradually adapt the effect of the gradient reversal layer because heart condition-dependent features are less prominent than domain-dependent features (see “Methods” section). The novelty here was the combination of alternate training (between standard 12-lead ECG images and mobile device-captured 12-lead ECG images) and its forward and backward propagation method, which changes dynamically during training epochs. Transfer learning was recently used to detect atrial fibrillation from 12-lead ECG vectors^13^. However, the approach was used to overcome limited quality labeled data, by shifting the training process from low-quality to high-quality data. The input was 12-lead ECG signals (which are not accessible in most clinics) and was tested on a single disease only.

One of the main limitations of using a deep learning approach is the need for many labeled samples. This limitation is a main bottleneck in the medical field because it requires many hours of work by experienced clinicians. ECG-Adversarial overcomes this limitation by its unique unsupervised transfer learning approach. The network reached high accuracy on mobile device-captured 12-lead ECG images when trained on unlabeled mobile device-captured 12-lead ECG images and scanned 12-lead ECG images. Note that due to effective augmentation methods that we used, even two subsequent frames from the same movie are different and sufficient to train the system.

When combining new unlabeled data of unseen formats with existing labeled, scanned 12-lead ECG images in a single training set, the network exhibited high accuracy in diagnosing the tested cardiac conditions (both morphological and arrhythmogenic). When the system was retrained on both rendered 12-lead ECG signals and 12-lead ECG mobile-captured images, its accuracy in interpreting rendered 12-lead ECG signals was further improved. While the dataset for retraining was very small, increasing its size will likely further increase the accuracy. Taken together, ECG-Adversarial is a robust system that can utilize new data gathered from different sources which use various formats and modalities.

Automated disease identification systems are faced with two main challenges; one is to handle different formats of 12-lead ECG images and the other is to extend the system to additional cardiac conditions. Our methods successfully handled several different formats of 12-lead ECG images that were not seen during training. To reach even higher accuracy on such unseen format, its unlabeled samples should be mixed with unlabeled samples from a known format used during training, and the system should be retrained. To train the system to identify a new cardiac condition, labeled samples of that condition must be added to the training set. Importantly, the new samples can use any 12-lead ECG image format and modality. Furthermore, because our approach is based on a generic architecture that is separately trained for binary identification of each disease, it simply requires samples that are labeled for the new condition. This method, tested here on data consisting of 12-lead ECG images, can potentially be extended to other medical image modalities such as MRI and CT scans.

Some ECG machines can provide a diagnosis. However, their average accuracy is 0.872, which is lower than that of our networks^2^. Note that these machines use signals rather than images or mobile device camera-captured images. Moreover, their algorithms are tuned to the specific properties of the machine type and thus are likely not compatible with machines produced by other vendors.

Our previous work^4^ showed slightly higher accuracy using signals rather than images as the analysis input. However, the accuracy of the method reported in this work was enhanced when images were used and reached the same accuracy for both modalities.

Others have attempted to generate automated ECG interpretation systems. Systems analyzing one-lead ECG (see for example^14^) cannot detect most morphological conditions. Systems analyzing 12-lead ECG suffer from the lack of sufficiently large datasets, a situation limiting their development to traditional, less effective ML methods that are based on human-crafted features^15^. More recent works target 12-lead ECG using state-of-the-art deep learning methods. For example, Ribeiro et al.^1^ used a huge 12-lead ECG signals dataset but did not test their method on images. Moreover, they detected only 6 diseases (out of 14 tested here). Similarly, Smith et al.^2^ achieved higher accuracy in interpreting 12-lead ECG recordings than the annotations of ECG machines in detecting non-specific conditions (major or no abnormality). However, their source format consisted of signals and not images.

To the best of our knowledge, PMCardio was the only application that applied a mobile AI cardiac condition-detection methods in a clinical environment^16^. While this work has clinical merit, PMCardio is compatible with only one format that was tested clinically, and sufficiently high accuracy was only achieved on atrial fibrillation and flutter conditions. Moreover, the approach requires accurate signal extraction from the captured image, which is a very challenging task. In our approach, cardiac conditions are detected directly from the image, with no need for signal extraction. Other works showed poor efficacy and poor accuracy in clinical conditions for testing available wearables (Apple Watch, AliveCor etc.) accuracy for atrial fibrillation (the most common arrhythmia)^17^.

Sane et al.^3^ digitized 12-lead ECG images and successfully extracted the signal even when the page was skewed; they detected myocardial infraction. Similarly, our previous work^4^ rendered 12-lead ECG signals into images for automated ECG interpretation of combinations of 8 cardiac conditions from tilted and skewed paper images. In contrast, here, we demonstrated high accuracy of automated ECG interpretation of real 12-lead ECG images that were captured by a mobile device. Our methods can deal with unlabeled formats and identify 14 cardiac conditions using a generic architecture that can be extended to more diseases and formats.

### Limitations

ECG-Adversarial was tested on six different unseen formats. Although the algorithm reached high accuracy, additional unseen formats will be needed to strengthen the performance claims. We showed that when meeting a new unseen format, the network can be retrained with unlabeled samples to improve results.

High accuracy was achieved when ECG-Adversarial was trained on mobile-captured 12-lead ECG images. Higher diversity (e.g., different formats, different population) of such samples will improve the robustness and performance of the system. Moreover, the mobile-device acquired 12-lead ECG images were generated in a synthetic manner. Using real-world mobile device captured images from many hospitals is necessary to generalize the approach.

“Explainability” indeed essential for translation of bench findings to clinical application and for building trust in AI applications. Yet, it is challenging to provide high-quality, explainability on mobile-captured 12-lead ECG images that can be easily applied in a clinical environment. Explainability methods designed for mobile-captured 12-lead ECG images will be necessary to correlate the features of the model proposed in this work and the features of the clinician's diagnostic process.

## Methods

### General approach

The main goal of this work was to enable identification of cardiac conditions from 12-lead ECG recording in the clinic. To do so using currently available technologies, it makes sense to digitize the plots that are the output of most ECG machines by taking their photos using mobile cameras (e.g., smartphones). Thus, a major milestone is to show that analysis of 12-lead ECG images taken in the clinic using a mobile device is feasible. Since no dataset of such images exists, this work used a two-step approach. We first used the large, labeled NYU dataset of high-quality scans of 12-lead ECG images (Fig. S4A) of various cardiac conditions (Fig. S4B) to train a deep neural network called ECG-Vanilla. ECG-Vanilla is a generic, binary classifier, with one feature extractor and one label predictor (Fig. 3). Note, that more than one cardiac condition might be present in the same image. To overcome this problem, we used a scalable approach that consisted of a set of binary classification networks, one per condition. Given an ECG image, once the inference process is performed on all the networks, we are left with a potential set of conditions.

However, as expected, ECG-Vanilla showed poor performance when tested on images from different distributions and/or different formats than that of the NYU dataset. Thus, we designed a neural network, called ECG-Adversarial, which uses a transfer learning approach, called domain adversarial training. ECG-Adversarial is a deep convolutional neural network that, in addition to the feature extractor and label predictor, also includes a domain classifier (Fig. 3). Similarly, to identify the presence of multiple conditions, we used the scalable method described above.

## Datasets

### NYU dataset

The "NYU School of Medicine Emergency Care Electrocardiogram (ECG) Database" (https://education.med.nyu.edu/ecg-database/app) consists of 79,226 scanned labeled ECGs associated with at least one out of 93 possible cardiac diagnoses, with a possibility of more than one disease label per image. The images were collected from patients who visited the NYU Langone Medical Center Perelman Emergency Department over a five-year period. Of note, 81,287 scanned labeled ECGs exist in the dataset, however, 2061 images were not available for download. A sample record from the dataset is presented in Fig. S4A.

Because deep learning methods are only applicable when a large dataset is available, we assembled subgroups of the same clinical conditions. The "ST changes" group included records labeled “ST changes—Nonspecific ST deviation”, “ST changes—Nonspecific ST deviation with T-wave change", "ST changes—Nonspecific T-wave abnormality” or "ST-T change—ventricular hypertrophy". “Right bundle branch block (RBBBs)” included records that were labeled as "Bundle-branch block—RBBB—incomplete" or "Bundle Branch Block—Right—RBBB". “ST elevation—myocardial infarction (STEMIs)” included records that were labeled as "STEMI—Anteroseptal", "STEMI—Anterior", "STEMI—Inferior or Inferolateral", "STEMI—Lateral", "STEMI—Posterior" or "STEMI—Right Ventricular". The network was not trained to identify cardiac conditions that had fewer than 828 records. In total, we obtained 14 disease categories for training. All records with a diagnosis that related to a specific category were labeled as "True" with regards to this category, whereas the remaining records were labeled as "False" with regards to that same category. A histogram of the distribution of records to the 14 categories is presented in Fig. S4B. The number of positive and negative samples of each of the 14 categories is presented in Table S5.

After an initial split of the data into development (85% of the population; 67,351 records) and holdout (15% of the population; 11,885 records) datasets, the development dataset was further divided into training (94% of the development set) and internal validation (6%) datasets.

### Mobile device-acquired unlabeled 12-lead ECG images

We printed 40 records from the NYU dataset. To avoid contamination of the training sets with samples from the test set (see details below), all printed records were from the training set only. We captured a video stream of each image. To create artifacts, we placed the printed page of each record next to a working ventilator connected to randomly vibrating thin films that were illuminated by a light source. The light introduced random occlusions and the turbulence of ventilator air flow caused the films to fluctuate rapidly, thereby introducing substantial artifacts in the frames (see Fig. S5). An example of two subsequent frames is presented in Fig. S6. Each individual frame was considered as a record in the unlabeled mobile device-acquired 12-lead images dataset. In total, this training dataset included 39,613 records.

The above 39,613 unlabeled mobile device frames were mixed with an additional 39,613 scanned images taken from NYU dataset (development), resulting in 79,226 samples in total. There was no data contamination on a patient level, separation of datasets into training/validation/testing was conducted at a patient level. Thus, the training set that was used for forward and backpropagation through the label predictor (NYU dataset) and the one that was used for forward and backpropagation through the domain classifier, were equal in size. In machine learning, the validation dataset is a sample of data used to provide an unbiased evaluation of a model fit while tuning model hyperparameters. Hyperparameter optimization was performed on ECG-Vanilla. Since the label predictor and feature extractor of ECG-Adversarial are to those of ECG Vanilla, there was no need for an internal validation dataset for the development of ECG-Adversarial, as the hyperparameters that are used for ECG-Adversarial, and ECG-Vanilla are the same.

### Mobile device-acquired labeled 12-lead ECG images

To generate a holdout testing dataset that includes data close to clinical conditions, we randomly chose 121 samples from the holdout NYU 12-lead ECG images dataset and captured a video stream of each. Each video was split to frames, as described for the mobile device-acquired 12-lead ECG images. In total, the holdout set consisted of 10,769 images that were labeled the same as the corresponding original image in the NYU dataset. The number of positive and negative samples in each of the 14 categories in the test set is presented in Table S6.

### Mobile device-acquired 12-lead ECG images of different formats

ECG samples were taken from different countries and continents, representing various populations. We printed 13 records from different sources, including one internal source. A video stream of each was captured^18–28^. Each video was split to frames, as described for the mobile device-acquired 12-lead ECG images. In total, this labeled dataset included 2700 records and was only used for testing. Six sample images are presented in Fig. S1. For each record, presence of a certain condition was labeled as "True" for the specific condition, whereas absence of the condition was labeled as "False". Therefore, all records were used for all categories, without data exclusion.

### Mix of mobile device-acquired 12-lead ECG images of different formats and NY format

This dataset was generated from a mix of datasets 2 and 4.

To include different formats, 20,000 of the original mobile device-acquired dataset images (dataset 2) were randomly replaced with images generated from video capture split to frames of 6 different ECG recordings in formats different from that use by the database 4.

### 12-lead ECG images rendered from signals

Using a method similar to that used in our previous study^4^, signal-based ECGs were artificially rendered from publicly available databases (Fig. S7). More specifically, 6,877 signal-based ECGs were artificially rendered from the “China Physiological Signal Challenge 2018”^29^, 21,799 signal-based ECGs were artificially rendered from the PTB-XL ECG dataset^30^ and 32,862 signal-based ECGs were artificially rendered from Ningbo First Hospital of Zhejiang University database^31^. The first 10 s of each record were used (2.5 s for each lead and 10 s for lead II). Training was performed with 5000 unlabeled records and the remaining 1877 records were used as the test set. Because this dataset included different diseases than those included in the NYU dataset (with a very small overlap), we focused on arrhythmogenic disorders (atrial fibrillation—AF and sinus bradycardia) and morphological disorders (left bundle branch block—LBBB and VPB-VPC).

A new training dataset was created by replacing 5000 images in the training dataset of mobile device-acquired 12-lead ECG images (dataset 2) with rendered 12-lead ECG images from signals. We experimented using two different test sets: the original test set of mobile device-acquired 12-lead ECG images (dataset 2) and a test set of 1,887 12-lead ECG images rendered from signals.

## Overview of the deep network model

DNNs were implemented using the Pytorch Framework with Python. The same basic architecture of the feature extractor was used for both ECG-Vanilla and ECG-Adversarial. In ECG-Vanilla, after reshaping the feature extractor to one dimensional vector, its output was used as an input to the fully connected label predictor, whereas in ECG-Adversarial, after flattening, the feature extractor output was used as input to both a fully connected label predictor and domain classifier (see Fig. 3).

In ECG-Adversarial, we placed an adaptive gradient reversal layer between the feature extractor and the domain classifier, to act as a pass-through during forward propagation but inverses the gradient sign and applies scaling during backpropagation. The gradient reversal layer ensures that the feature distributions over the two domains are made as indistinguishable as possible to the domain classifier, resulting in selection of domain-invariant features only.

Similar to Ganin^9^, we used a dynamic domain adversarial training model called lambda, to control the impact of the domain classifier inverse gradients during backpropagation. We found that constant lambda performed poorly with physiological signals because heart condition-dependent features are less prominent than domain-dependent ones, with the ratio between the two changing during the training process. To overcome the difference in the convergence rate (i.e., difference in gradient values) between the label predictor and the domain classifier, we used dynamic lambda suitable for compensation of differences between the two modalities (perfectly scanned images vs. mobile captured images), as described in Eq. (1).

1 $$\lambda =0.85{e}^{5.5p} ,p=\frac{b+E*{N}_{B}}{{N}_{Batches}*{N}_{Epoch}},$$

where b is a batch index within an epoch, E is the epoch index, ${N}_{Batches}$ is the number of batches in an epoch, and ${N}_{Epoch}$ is the epoch number.

**Figure 5:**
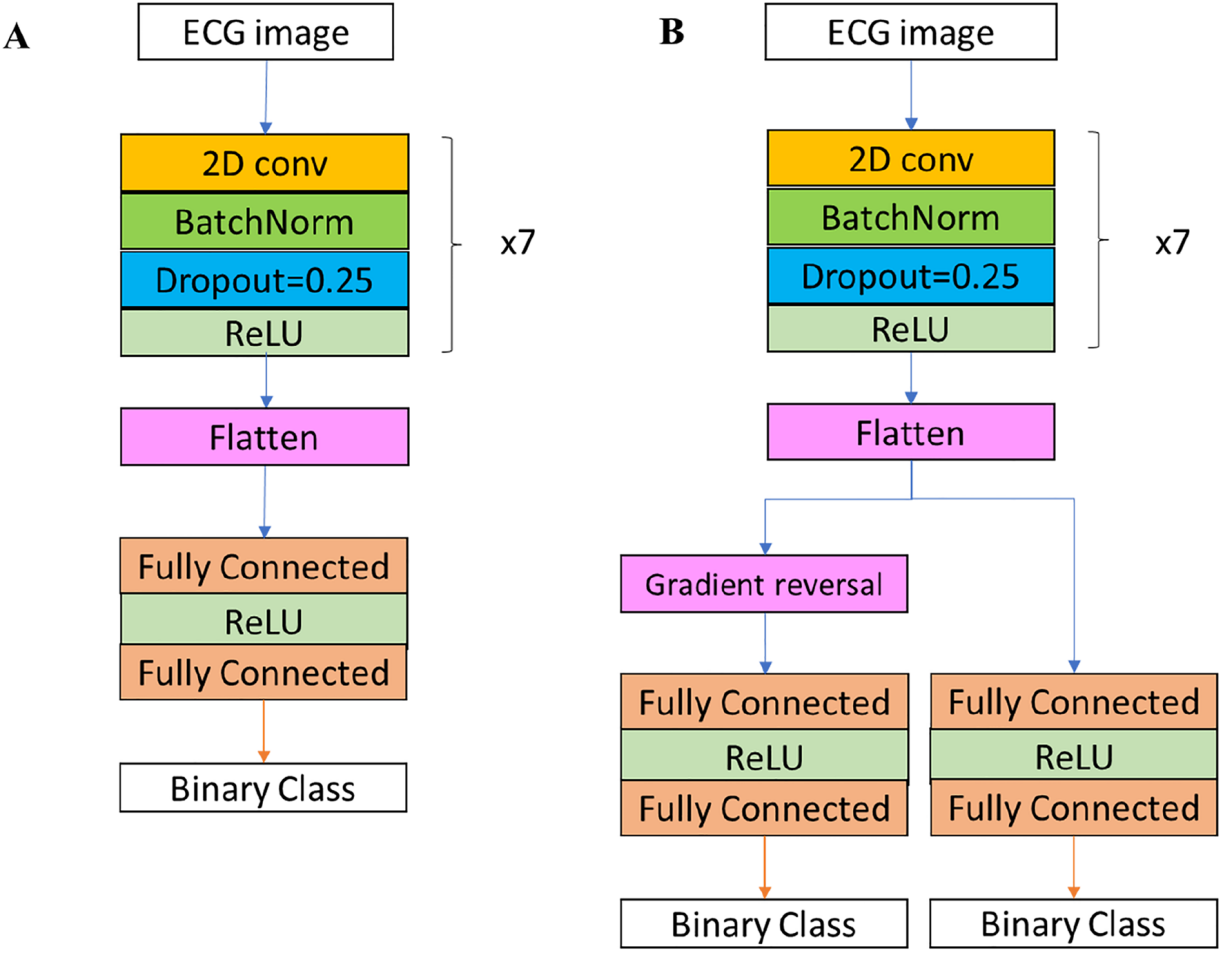
Networks architecture. (**A**) Network architecture for 12-lead ECG image classification, ECG-Vanilla. Input images were of size 880 × 1650 × 3 color values. The input data enters a convolution layer with a stride step of [1, 2, 2, 2, 2, 2, 2] for each of the 7 blocks, respectively. Next is a batch normalization layer in which the batch distribution is normalized. The dropout layer randomly deletes a fraction of the network edges with a given probability during the training, to improve robustness^32^. We used a dropout probability of 0.25. Next, the 3D convolution output is flattened, is passed through a fully connected classification layer, and serves as a threshold for the outcome. Each of the resulting networks is trained on a single condition and generates a binary output for each input: existence or absence of a certain cardiac disorder. Convolution kernel size was 7 × 7. Hidden layers size was [8,16,32,64,128,256,512] for each of the convolutional layers, respectively. (**B**) Architecture of the 12-lead ECG image classification network, ECG-Adversarial. Input images (either image or mobile device-acquired 12-lead ECG images) were of the size 880 × 1650 × 3 color values. Feature extractor, convolution layer, normalization layer, dropout layer, convolution kernel size, hidden layers size, and fully connected blocks of ECG-Adversarial, were all as in ECG-Vanilla. The 3D convolution output is flattened, is passed through a fully connected classification layer, and serves as a threshold for the outcome for label predictor and in parallel goes through a gradient reversal layer and then a fully connected classification layer and serves as a threshold for the outcome for domain classifier. As in ECG-Vanilla, each of the resulting networks is trained on a single condition and generates a binary output for each input: existence or absence of a certain cardiac disorder. As in ECG-Vanilla, the 3D convolution output is flattened, and is passed through a fully connected classification layer and threshold to yield the outcome.

Both ECG-Vanilla and ECG-Adversarial networks were built using stacked blocks of convolutional, batch normalization and dropout (Fig. 5). Both networks used a linear rectifier (ReLU) as their activation function. Hyperparameters (batch size, initial learning rate, number of nodes in the fully connected layers, and number of convolutional layers) were adjusted during training to obtain the optimal model. The initial learning rate was 4e − 5 for both networks. Batch size during training was 50 for both networks.

## Training process

Both networks were trained using the Adam optimizer^33^, with the default parameters β_1_ = 0.9 and β_2_ = 0.999, and a binary cross-entropy as the loss function. We tested Adam optimizer learning rates between 10^–3^ and 10^–6^ and selected 4*10^–5^. The internal validation dataset was used to manually tune the hyperparameters and to select the optimal model. Each time the models reached their peak accuracy on the 12-lead ECG images holdout dataset (in the case of ECG-Adversarial, also when reaching 40–60% accuracy on the holdout dataset of mobile device-acquired 12-lead ECG images), a snapshot of the state of the system was taken (namely, a checkpoint). To avoid entering the overfitting stage, network training was discontinued when the performance on the test set ceased to improve for 5 consecutive epochs. Note that we had two test sets for model training—one that included NYU dataset samples only, which was used for accuracy assessment of ECG-Vanilla and of the label predictor of ECG-Adversarial, and a second that was an equal mix of NYU dataset images and mobile device-acquired 12-lead ECG images (for performance assessment of the domain classifier). To save the model state (checkpoint) for ECG-Adversarial, two conditions were required: (1) the label predictor should yield the best heart condition detection result, and (2) the domain classifier accuracy should be between 40 and 60% (approximately equal to "random guess"), indicating that the net is agnostic to domain type.

Data augmentation is a technique used in machine learning for generalization, to improve model accuracy, and to control overfitting. Augmentation is performed by perturbation of the original dataset, while preserving the original label of the newly generated samples^34^. Augmentation was applied using the Torchvision function “RandomPerspective”, which performs a random perspective transformation on a given image with a given probability. Degree of distortion was 0.15, and probability of a certain image to undergo transformation was 0.8. Note that augmentation was only applied on the NYU dataset, because mobile device-acquired 12-lead ECG images are already inherently augmented.

Deep network implementation was performed in Python, using the PyTorch framework (version 1.7.1). In addition, torchvision (version 0.8.2), numpy (version 1.19.5), opencv (version 4.5.1.48), pandas (version 1.1.5) and pillow (version 8.1.0) libraries were used. The training server consisted of Intel(R) Xeon(R) Gold 6230 @ 2.1 GHz. RAM: 500 GB. GPU: GeForce RTX 3090 (of NVIDIA). Cuda version was 11.0. OS: Ubuntu 18.04.5 LTS.

For ECG-Vanilla, training time was about 30 min per epoch; a maximum of 60 epochs were used, unless an early stopping condition was reached (i.e., 5 epochs in which training loss is getting smaller but test loss is not). For ECG-Adversarial, training time was 45 min per epoch; a maximum of 60 epochs were used, with early stopping conditions similar to the case of ECG-Vanilla. For both networks, forward-propagation through the DNN took about 1 s.

The actual learning rate used was selected in such a way that the learning process would be effective, i.e., large enough to converge with reasonable speed but small enough to sustain the learning process and avoid divergent behavior. We tested different learning rates for several epochs to find one suitable for our needs. Selected hyperparameters included a batch size of 50 for both networks.

The NYU dataset is highly imbalanced. To improve the balance of classes in both the training and test dataset, rare categories were oversampled to reach a similar distribution for all disorders.

To train the ECG-Adversarial, we randomly initialized all weights and biases of the feature extractor, label predictor and domain classifier. For the first 5 epochs, we drew a minibatch from the NYU dataset, forward propagated it through the network, then backpropagated it through the label predictor and updated the label predictor and feature extractor weights and biases. During the next epochs, we first drew a minibatch from the NYU dataset and forward propagated it through the network to the label predictor. Then, we calculated binary cross-entropy loss of the label predictor, backpropagated through the label predictor and updated the label predictor and feature extractor weights and biases. Afterwards, we drew a minibatch which was a random mix of samples from the NYU dataset and mobile device-acquired 12-lead ECG image dataset. This minibatch was forward-propagated through the feature extractor, gradient reversal layer (without applying it during forward-propagation) and domain classifier. Then, binary cross-entropy loss of the domain classifier was calculated. When backpropagating from the domain classifier, a gradient reversal layer was applied with adaptive coefficient $\uplambda$ (see formula (1)) and weights and biases of a feature extractor and domain classifier were adjusted. These steps were repeated until the maximum number of epochs or the early stopping condition was reached.

## Model evaluation and statistical methods

For each condition, a binary classifier (condition present or absent) was designed with an output P, where P was in the range of 0–1. P threshold was deduced from the ROC curve (Table S7).

The following metrics were calculated at binary decision thresholds for every cardiac condition, to assess performance of ECG-Vanilla network (tested on NYU dataset) and ECG-Adversarial network (tested on labeled mobile device-acquired 12-lead ECG images) on the hidden set:

2 $$True\, positive:\,\, TP=\frac{\#\, correctly \,detected \,disease\, occurences }{\#\,episodes \,with\, presense \,of\, the \,disease}$$

3 $$True \,negative: \,\,TN=\frac{\# \,correctly\, detected \,disease\, absense }{\#\, episodes \,with\, absense \,of\, the\, disease}$$

4 $$False \,negative: \,FN=\frac{\#\, misdetected\, disease\, occurences }{\#\,episodes \,with \,presense\, of\, the\, disease}$$

5 $$False \,positive: \,FP=\frac{\#\, incorectly \,classified\, episodes }{\# \,episodes\, with \,absense\, of \,the \,disease}$$

6 $$Sensitivity:\, TPR=\frac{TP}{TP+FN}$$

7 $$Specificity: \,TNR=\frac{TN}{TN+FP}$$

8 $$Precision \,or \,positive\, predictive \,value: \,PPV=\frac{TP}{TP+FP}$$

9 $$Negative \,predictive \,value:\, NPV=\frac{TN}{TN+FN}$$

10 $$Accuracy:\, ACC=\frac{TP+TN}{TP+TN+FP+FN}$$

11 $${F}_{1}=\frac{2*TP}{2*TP+FP+FN}$$

### Supplementary Information

Supplementary Information.

## Data Availability

The data analysis code, and a link to the datasets will be freely available on GitHub following publication of the paper (https://technionmail-my.sharepoint.com/:f:/g/personal/yyaniv_technion_ac_il/Eui5xubMPdxJnT1v0ohp4QQBIebH7pb1CW89mH5TX0znbA?e=HKHseq). Please contact Vadim Gliner vadim.gliner@gmail.com.
