# Supplementary material for: Using domain adaptation for classification of healthy and disease conditions from mobile-captured images of standard 12-lead electrocardiograms
Source: Sci Rep. 2023 Aug 28;13:14023. doi: 10.1038/s41598-023-40693-6 (PMC10462630; doi:10.1038/s41598-023-40693-6)
Supplement: Supplementary file 1 — Supplementary Information. [file 41598_2023_40693_MOESM1_ESM.docx]

**Using domain adaptation for classification of healthy and disease conditions from mobile-captured images of standard 12-lead electrocardiograms**

**Supplement**

**Gliner et al. Mobile-captured ECG classification**

Vadim Gliner^1^, Vladimir Makarov^2^, Arutyun I. Avetisyan^3^,

Assaf Schuster^1^ and Yael Yaniv^4^

^1^Computer Science Department, Technion-IIT, Haifa, Israel

^2^System Programming Lab, Novgorod State University, Russia

^3^Ivannikov Institute for System Programming of the Russian Academy of Sciences, Russia

^4^Laboratory of Bioenergetic and Bioelectric Systems, Biomedical Engineering Faculty, Technion-IIT, Haifa, Israel

**^*^Correspondence:**

Yael Yaniv, PhD
Laboratory of Bioenergetic and Bioelectric Systems, Biomedical Engineering Faculty, Technion—IIT, Haifa

Email: [yaely@bm.technion.ac.il](mailto:yaely@bm.technion.ac.il)
Phone: 972-4-8294124
Fax: 972-4-8294599


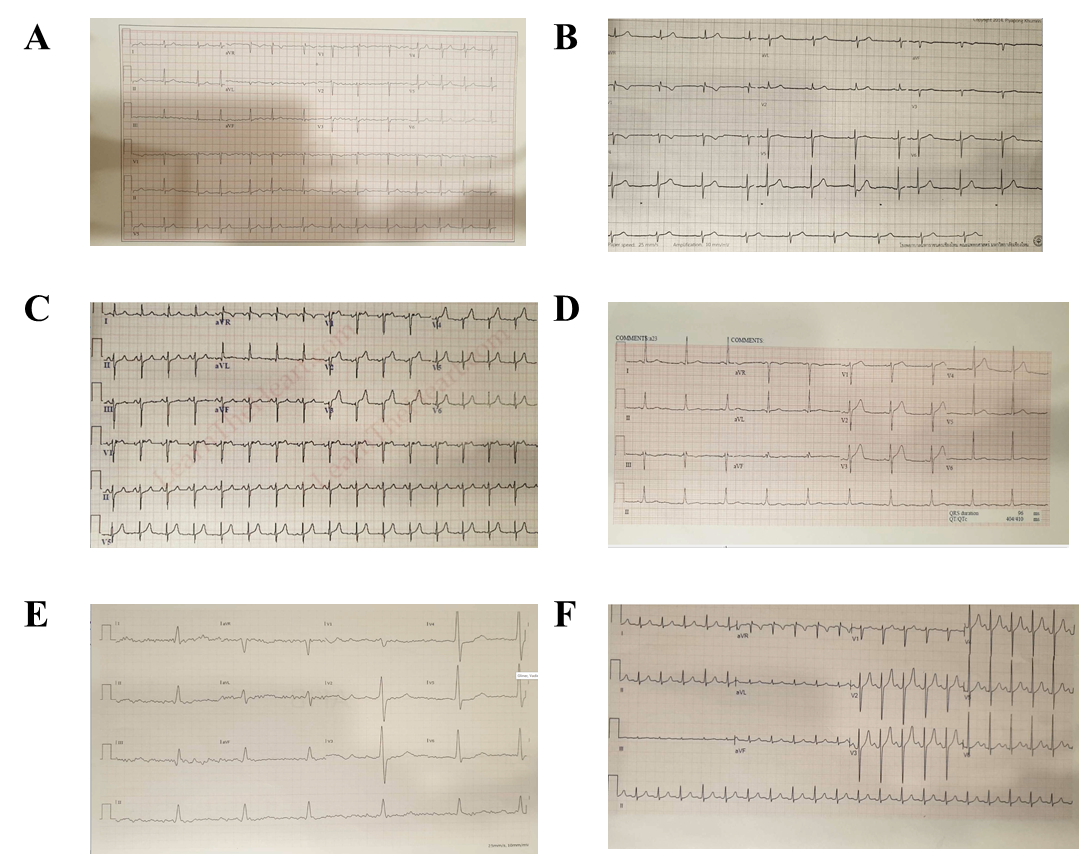


**Figure S1:** Six sample 12-lead ECG plots of different formats captured by a mobile camera and included in a test set. (A) Atrial fibrillation record from USA, (B) left axis deviation and sinus bradycardia record from India, (C) left-axis deviation record from USA, (D) left-axis deviation record from USA, (E) atrial fibrillation record from Velykiy Novgorod, Russia, and a (F) sinus tachycardia record from USA. Note that the formats differ in background color (B vs. D), number of long leads on the page (one long lead in F vs. three long leads in C), and page aspect ratio (C vs. D). In addition, artifacts like occlusions are more prominent on some (e.g., A).

**Figure S2:** The receiver operation characteristic area under the curve (ROC-AUC) of ECG-Adversarial when analyzing various formats that the networks did not see during training. The network was tested on 13 different 12-lead ECG recordings from 6 different unseen formats that were printed, filmed by a smartphone camera, and split to images (in total 2700 images). The ECG recordings were collected from patients from three different continents. (A) Atrial fibrillation, 1.0, (B) sinus bradycardia, 0.99, (C) left-axis deviation, 0.99, (D) left ventricular hypertrophy, 1.0 and (E) sinus tachycardia, 0.94.

**Figure S3:** The receiver operation characteristic area under the curve (ROC-AUC) of ECG-Adversarial on samples from various unseen formats, after the networks were fed samples from these formats during training. Tested on the same 13 records as in Figure S2 (in total 2700 images), which were of six different formats and recorded in three different continents. (A) Atrial fibrillation, 1.0, (B) sinus bradycardia, 1.0, (C) left-axis deviation, 1.0, (D) left ventricular hypertrophy, 0.99 and (E) sinus tachycardia, 1.0.


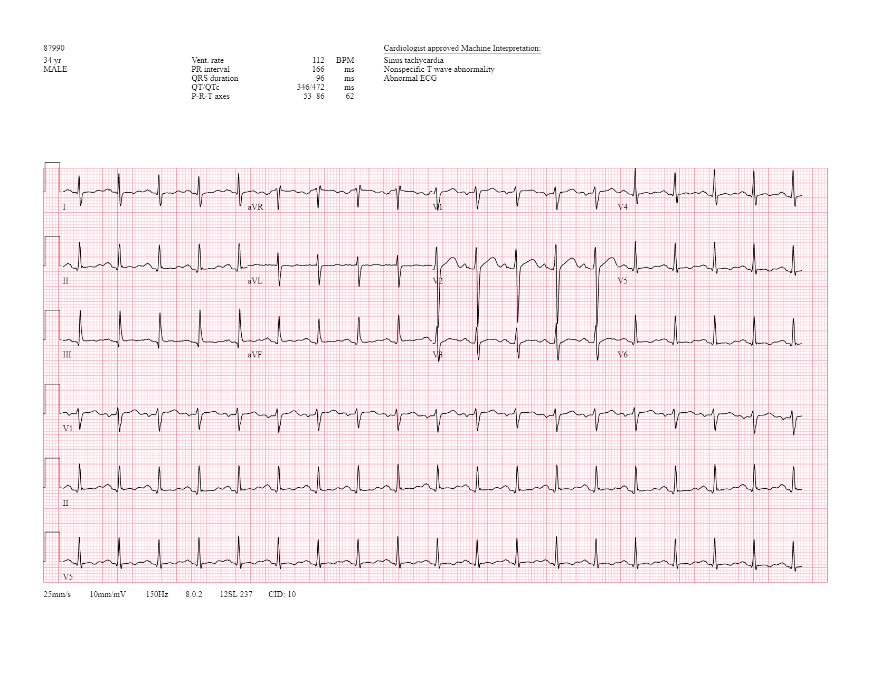


**A**

**B**

**Figure S4:** (A) Sample record from the NYU database and (B) histogram of 14 most prevalent cardiac disease categories in the NYU dataset.


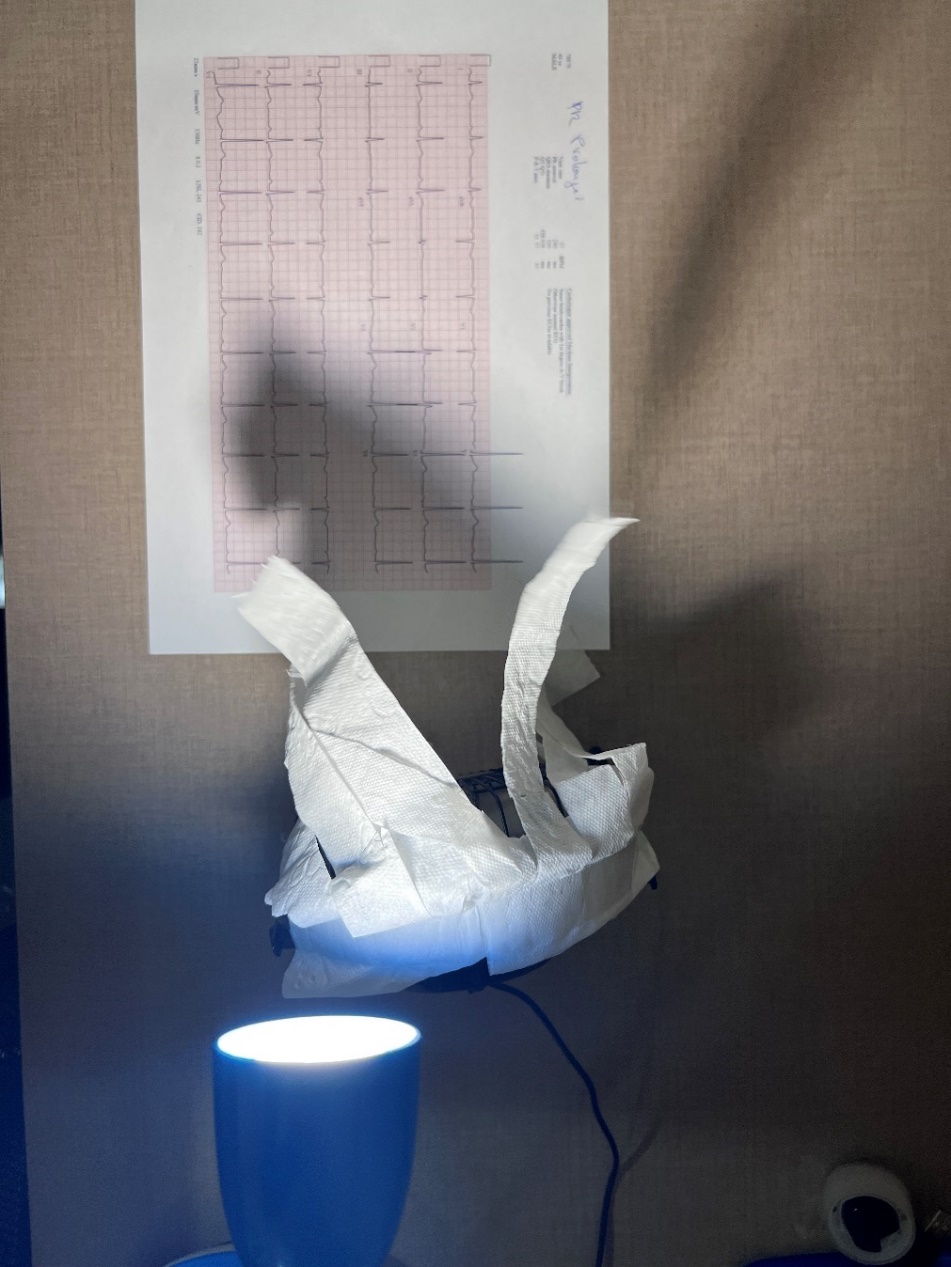


**Figure S5:** Setup of a working ventilator connected to randomly vibrated thin films that were illuminated by a light source. The light introduced random occlusions and the turbulence of ventilator air flow caused the films to fluctuate rapidly, thereby introducing substantial artifacts in the frames. This setup was used to introduce random artifacts on 12-lead ECG images acquired by a mobile device.


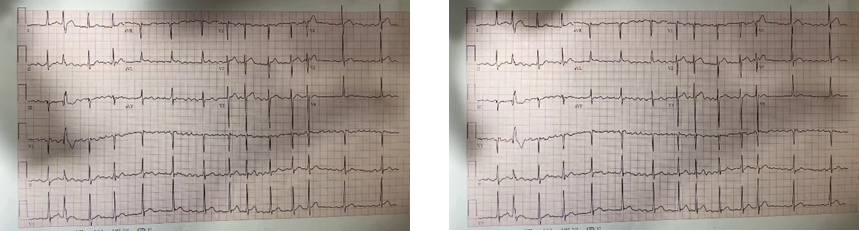


**Figure S6:** Two sample frames generated from one of the records in the NYU database using our ventilator setup. Images were captured by a mobile device. These types of images were used in the domain-adversarial training of ECG-Adversarial.


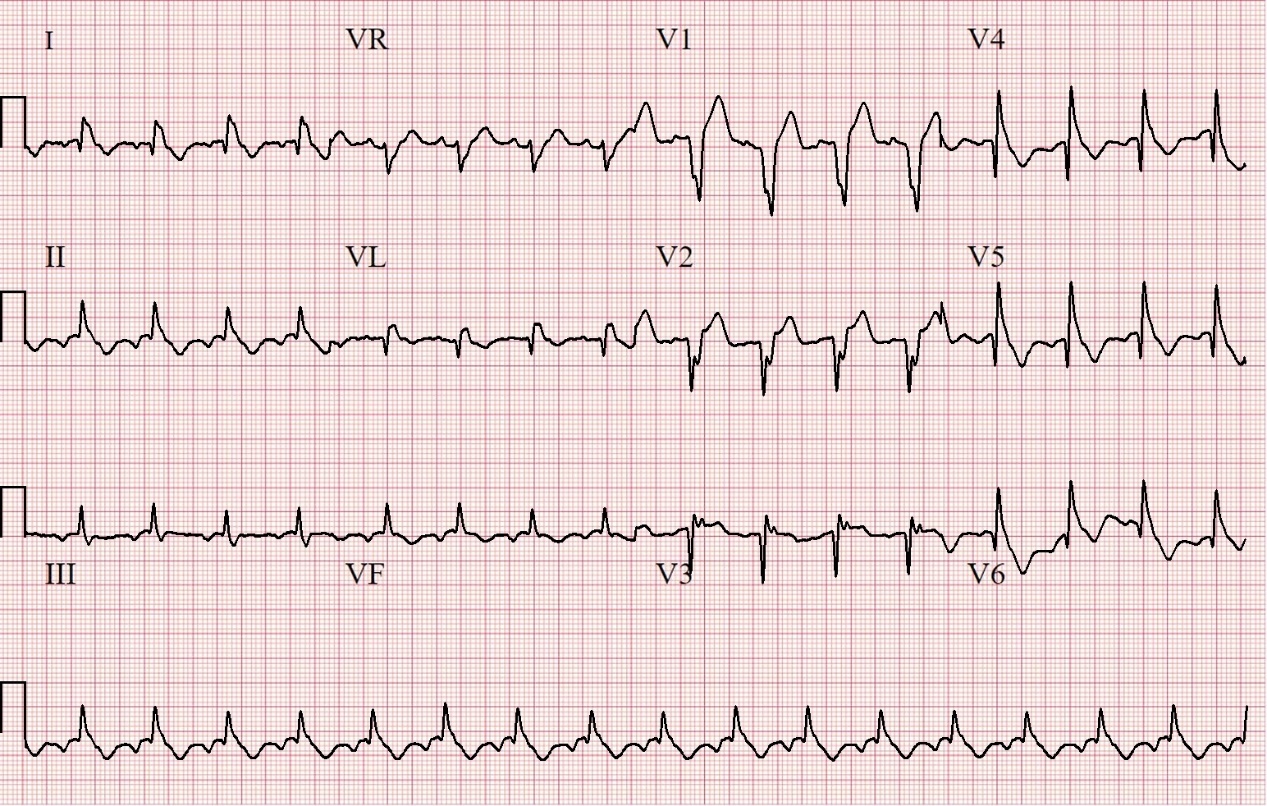


**Figure S7:** Rendering of record A0001 of 12-lead ECG digitized vectors. Labeled in the Chinese physiological challenge database^28^ as “Bundle Branch Block - Right – RBBB”.

**Table S1:** Performance measurements of ECG-Vanilla on a hidden test set with the same distribution as that of the training.

| **Category** | **Accuracy** | **Sensitivity** | **Specificity** | **Precision (positive predictive value)** | **Negative predictive value** |
| --- | --- | --- | --- | --- | --- |
| **Sinus tachycardia** | 0.99 | 0.99 | 0.99 | 0.99 | 0.99 |
| **Left atrial enlargement** | 0.95 | 0.95 | 0.94 | 0.94 | 0.95 |
| **ST changes - Nonspecific** | 0.83 | 0.83 | 0.83 | 0.82 | 0.83 |
| **Left ventricular hypertrophy** | 0.96 | 0.97 | 0.95 | 0.95 | 0.97 |
| **Sinus bradycardia** | 0.98 | 0.98 | 0.98 | 0.98 | 0.98 |
| **Sinus arrhythmia** | 0.92 | 0.92 | 0.91 | 0.91 | 0.92 |
| **STEMIs (ST elevation due to myocardial infarction)** | 0.87 | 0.88 | 0.86 | 0.86 | 0.88 |
| **Left-axis deviation** | 0.95 | 0.95 | 0.94 | 0.94 | 0.95 |
| **RBBBs (Right bundle branch block (complete and incomplete)** | 0.95 | 0.95 | 0.95 | 0.95 | 0.95 |
| **Normal variant** | 0.91 | 0.93 | 0.9 | 0.9 | 0.93 |
| **Atrial fibrillation** | 0.98 | 0.98 | 0.98 | 0.98 | 0.98 |
| **Ventricular premature complex(es) - VPB - VPC** | 0.93 | 0.93 | 0.94 | 0.94 | 0.93 |
| **QT interval - Prolonged** | 0.88 | 0.88 | 0.88 | 0.87 | 0.89 |
| **Bundle branch block - Left - LBBB** | 0.99 | 0.99 | 0.99 | 0.99 | 0.99 |

**Table S2:** Performance measurements of ECG-Vanilla on a hidden test set with the same distribution as that of the training vs. ResNet18.

| **Category** | **ROC-AUC**  **ECG-Vanilla** | **ROC-AUC**  **ECG-ResNet18** |
| --- | --- | --- |
| Atrial fibrillation | 0.98 | 0.98 |
| Premature ventricular contraction | 0.93 | 0.92 |
| Left axis deviation | 0.95 | 0.94 |
| Left bundle branch block | 0.99 | 0.99 |
| Sinus tachycardia | 0.99 | 0.99 |
| Left atrial enlargement | 0.95 | 0.95 |
| ST changes | 0.83 | 0.84 |
| Left ventricular hypertrophy | 0.96 | 0.95 |
| Sinus bradycardia | 0.98 | 0.98 |
| Sinus arrhythmia | 0.92 | 0.92 |
| ST elevation, myocardial infarction | 0.87 | 0.85 |
| Right bundle branch block | 0.95 | 0.95 |
| Normal variant | 0.91 | 0.9 |
| QT Interval, prolonged | 0.88 | 0.88 |
| **Average** | **0.94** | **0.93** |

**Table S3:** Performance measurements of ECG-Adversarial on the hidden test set

| **Category** | **Accuracy** | **Sensitivity** | **Specificity** | **Positive predictive value** | **Negative predictive value** | **F1** |
| --- | --- | --- | --- | --- | --- | --- |
| **Sinus tachycardia** | 0.78 | 0.72 | 0.86 | 0.88 | 0.69 | 0.79 |
| **Left atrial enlargement** | 0.89 | 0.92 | 0.86 | 0.88 | 0.9 | 0.9 |
| **ST changes – Nonspecific)** | 0.83 | 0.85 | 0.82 | 0.83 | 0.84 | 0.84 |
| **Left ventricular hypertrophy** | 0.85 | 0.88 | 0.82 | 0.84 | 0.86 | 0.86 |
| **Sinus bradycardia** | 0.87 | 0.89 | 0.85 | 0.87 | 0.87 | 0.88 |
| **Sinus arrhythmia** | 0.78 | 0.84 | 0.72 | 0.76 | 0.81 | 0.8 |
| **STEMIs (ST elevation due to myocardial infarction** | 0.81 | 0.79 | 0.83 | 0.84 | 0.77 | 0.81 |
| **Left-axis deviation** | 0.83 | 0.87 | 0.78 | 0.81 | 0.85 | 0.84 |
| **RBBBs (Right bundle branch block** | 0.91 | 0.91 | 0.92 | 0.92 | 0.9 | 0.91 |
| **Normal variant** | 0.93 | 0.98 | 0.87 | 0.89 | 0.97 | 0.93 |
| **Atrial fibrillation** | 0.91 | 0.92 | 0.89 | 0.91 | 0.9 | 0.91 |
| **Ventricular premature complex(es) - VPB – VPC** | 0.77 | 0.81 | 0.71 | 0.8 | 0.72 | 0.81 |
| **QT interval – Prolonged** | 0.89 | 0.88 | 0.89 | 0.91 | 0.86 | 0.9 |
| **Bundle branch block - Left – LBBB** | 0.88 | 0.87 | 0.89 | 0.91 | 0.84 | 0.89 |

**Table S4:** Performance of original and retrained networks on a hidden test set of mobile device-acquired 12-lead ECG images and rendered images. Original - trained on mobile device-acquired 12-lead ECG images. Retrained - trained on a database consisting of both 12-lead ECG images with smartphone camera acquisition distortion and images that were generated from signals.

| Category | Original **ROC-AUC - With augmentation, dropout=0.25, epoch of start training the domain head =5** assessment on hidden test set from *12-lead ECG images with mobile device acquisition database* | Original **ROC-AUC - With augmentation, dropout=0.25, epoch of start training the domain head =5** assessment on hidden test of artificially rendered signals | Retrained **ROC-AUC - With augmentation, dropout=0.25, epoch of start training the domain head =5** assessment on hidden test set from *12-lead ECG images with mobile device acquisition database* | Retrained **ROC-AUC - With augmentation, dropout=0.25, epoch of start training the domain head =5** assessment on hidden test of artificially rendered signals |
| --- | --- | --- | --- | --- |
| AF (CPSC) | 0.96 | 0.81 | 0.87 | 0.9 |
| AF (PTB-XL & Ningbo) | 0.96 | 0.95 | Not tested | 0.95 |
| Bundle branch block - Left (LBBB) (CPSC) | 0.94 | 0.83 | 0.92 | 0.94 |
| Bundle branch block - Left (LBBB) (PTB-XL & Ningbo) | 0.94 | 0.93 | Not tested | 0.95 |
| PVC(CPSC) | 0.82 | 0.95 | Not tested | 0.97 |
| SB(CPSC) | 0.95 | 0.95 | Not tested | 0.96 |
| **Mean** | **0.93** | **0.90** | **0.90** | **0.95** |

**Table S5**: Number of positive and negative samples of each of the 14 categories of cardiac conditions in the NYU dataset

| **Disease** | **True** | **False** |
| --- | --- | --- |
| ST changes – Nonspecific | 17629 | 61607 |
| STEMIs | 11889 | 67347 |
| Sinus tachycardia | 10190 | 69046 |
| Left atrial enlargement | 10099 | 69137 |
| Left ventricular hypertrophy | 8069 | 71167 |
| Sinus bradycardia | 7213 | 72023 |
| RBBBs | 6494 | 72742 |
| Sinus arrhythmia | 5292 | 73944 |
| Left-axis deviation | 4562 | 74674 |
| Normal variant | 3118 | 76118 |
| Atrial fibrillation | 3055 | 76181 |
| Ventricular premature complex(es) - VPB – VPC | 3001 | 76235 |
| QT interval – Prolonged | 2865 | 76371 |
| Bundle branch block - Left – LBBB | 828 | 78408 |

**Table S6**: Number of positive and negative samples for the 14 categories of cardiac conditions in a test set taken from mobile device-acquired 12-lead ECG images dataset

| **Category** | **Number of Positive Samples** | **Number of Negative Samples** |
| --- | --- | --- |
| ST changes | 445 | 10324 |
| STEMIs | 1780 | 8989 |
| Sinus tachycardia | 3471 | 7298 |
| Left atrial enlargement | 1246 | 9523 |
| Left ventricular hypertrophy | 979 | 9790 |
| Sinus bradycardia | 1335 | 9434 |
| RBBBs | 1246 | 9523 |
| Sinus arrhythmia | 356 | 10413 |
| Left-axis deviation | 1513 | 9256 |
| Normal variant | 356 | 10413 |
| Atrial fibrillation | 1869 | 8900 |
| Ventricular premature complex(es) - VPB - VPC | 2670 | 8099 |
| QT interval - Prolonged | 1513 | 9256 |
| Bundle branch block - Left - LBBB | 1869 | 8900 |

**Table S7**: Classification thresholds of the 14 categories based upon model operating point from the ROC curve

| **Category** | **Model operating point**  **(Decision threshold)** |
| --- | --- |
| ST changes | 0.9180 |
| STEMIs | 0.0025 |
| Sinus tachycardia | 0.4055 |
| Left atrial enlargement | 0.4507 |
| Left ventricular hypertrophy | 0.0236 |
| Sinus Bradycardia | 0.2082 |
| RBBBs | 0.7506 |
| Sinus arrhythmia | 0.0431 |
| Left-axis deviation | 0.8942 |
| Normal variant | 0.1764 |
| Atrial fibrillation | 0.986 |
| Ventricular premature complex(es) - VPB - VPC | 0.5909 |
| QT interval - Prolonged | 0.2049 |
| Bundle branch block - Left - LBBB | 0.3363 |
